# Vocalization-associated respiration patterns: thermography-based monitoring and detection of preparation for calling

**DOI:** 10.1242/jeb.243474

**Published:** 2022-03-10

**Authors:** Vlad Demartsev, Marta B. Manser, Glenn J. Tattersall

**Affiliations:** 1 Department of Biology, University of Konstanz, 78464 Konstanz, Germany; 2 Department for the Ecology of Animal Societies, Max Planck Institute of Animal Behavior, 78467 Konstanz, Germany; 3 Kalahari Research Centre, 8467 Van Zylsrus, Northern Cape, South Africa; 4 Department of Evolutionary Biology and Environmental Studies, University of Zurich, 8057 Zurich, Switzerland; 5 Interdisciplinary Center for the Evolution of Language, University of Zurich, 8057 Zurich, Switzerland; 6 Biological Sciences, Brock University, St. Catharines, ON, Canada, L2S 3A1

**Keywords:** Acoustic signalling, Respiration monitoring, Thermography, Vocal emission

## Abstract

Vocal emission requires coordination with the respiratory system. Monitoring the increase in laryngeal pressure, which is needed for vocal production, allows detection of transitions from quiet respiration to vocalization-supporting respiration. Characterization of these transitions could be used to identify preparation for vocal emission and to examine the probability of it manifesting into an actual vocal production event. Specifically, overlaying the subject's respiration with conspecific calls can highlight events of call initiation and suppression, as a means of signalling coordination and avoiding jamming. Here, we present a thermal imaging-based methodology for synchronized respiration and vocalization monitoring of free-ranging meerkats. The sensitivity of this methodology is sufficient for detecting transient changes in the subject's respiration associated with the exertion of vocal production. The differences in respiration are apparent not only during the vocal output, but also prior to it, marking the potential time frame of the respiratory preparation for calling. A correlation between conspecific calls with elongation of the focal subject's respiration cycles could be related to fluctuations in attention levels or in the motivation to reply. This framework can be used for examining the capability for enhanced respiration control in animals during modulated and complex vocal sequences, detecting ‘failed’ vocalization attempts and investigating the role of respiration cues in the regulation of vocal interactions.

## Abstract (precis)

**Summary:** A thermography-based methodology for estimating breathing traces in free-ranging meerkats detects changes in respiration associated with the preparation and with the production of vocal signals by combining respiration monitoring with audio recordings.

## INTRODUCTION

Vocal communication is a multi-stage process. The basic vocal production-and-reply exchange can be described as: (1) the reception and neural translation of an audible signal; (2) the informational integration (Belin et al., 2000); (3) the activation of a behavioural response (Chen et al., 2009); (4) the motor preparation of the vocal apparatus (Winkworth et al., 1995); and finally, (5) the sound emission. However, most current research on vocal exchanges focuses mainly on the first and the last stages – the acoustic stimulus and its effect on the receiver's behaviour (e.g. the presence or absence of a vocal response; Herbinger et al., 2009; Schulz et al., 2008; Seyfarth and Cheney, 2003; Sugiura, 1998). Such an approach creates a binary link between stimulus and response, largely ignoring potentially intervening factors that might modify the communication event. Monitoring neural (Hage and Nieder, 2013) and/or physiological processes in the gap between a stimulus and a response would enable examination of the communication events in more detail. For example, perceived stimuli being ignored as not relevant or not triggering a vocal response as a tactical decision (e.g. confrontation avoidance) will register on a neural/physiological level and could be distinguished from an undetected stimulus, despite both of these conditions not manifesting into a behavioural reaction. Additionally, quantifiable physiological changes, triggered by a vocal input and preceding observable behaviour, could reflect modulation of response motivation and allow detection of signal intensity and type-dependent response thresholds. Furthermore, monitoring the timing and the dynamics of physiological changes, at the gap between receiving and responding to a call, might allow estimation of the extent of preparation for vocal emission (Rochet-Capellan and Fuchs, 2014), and perhaps the levels of volitional control over initiation and duration of vocalizations.

Because communication is a socially driven process (Blumstein and Armitage, 1997), observing it under natural group settings is needed to generate insights into its ecologically relevant dynamics. While recording the neural activity throughout stimuli perception (Aubie et al., 2014) and vocal production (West and Larson, 1993; Wild, 1997) is possible, it is not easily implemented outside laboratory conditions. Monitoring of physiological processes, on the other hand, is less invasive and more suitable for field-based studies (Elliott, 2016). One candidate physiological process that is directly related to vocal emission is respiration (Riede, 2011; Smotherman et al., 2006). The mechanism of vocal production relies on a flow of gases within the respiratory tract, generating sufficient subglottal pressure for laryngeal tissue vibration and emission of sound (Herbst, 2016). The orientation of the vocal folds in most terrestrial mammals allows efficient sound production on the expiration phase. Phonation by an inspiratory air stream is also possible, but rarely occurs (Fitch, 2006). The duration of continuous vocalization and the rate of vocal unit production is limited by the caller's lung capacity and by the thoracic muscle activity, the latter aiding in maintaining subglottal pressure beyond the point of elastic lung recoil (MacLarnon and Hewitt, 1999). The pressure requirements of vocal production, call structure and duration, introduce alterations to the ‘quiet’ respiration pattern (Häusler, 2000; Hernandez et al., 2017).

Studies of respiration patterns in the context of vocal communication have traditionally focused on human conversations. Stereotypic breathing changes have been detected before the initiation of speaking turn, allowing better timing of speaker exchange and overlap avoidance. Speaking duration is positively correlated with the inspiration amplitude, potentially indicating early planning of the speaking turn. Additionally, breathing patterns are used by the counterparts in a conversation as cues indicating intention to speak (MacLarnon and Hewitt, 1999; McFarland, 2001; Rochet-Capellan and Fuchs, 2014). Animals have been claimed to have less sophisticated control over their respiration in the context of vocal emission (Maclarnon and Hewitt, 2004; MacLarnon and Hewitt, 1999). However, several studies have demonstrated a disruption of the respiration cycle after (Laplagne, 2018) and before vocal onset (Häusler, 2000; Hernandez et al., 2017; Riede et al., 2020) in animals, suggesting that claims of complex vocalization-correlated respiratory movements (VCRMs) being uniquely human (Häusler, 2000) are not well justified.

Here, we establish a proof-of-concept methodology for remote respiration monitoring in a free-ranging animal, as a tool for tracing VCRMs. Additionally, we demonstrate the ability to characterize changes in respiration preceding vocal production and to detect the extent of the preparation phase that the individual needs to produce a call. Additionally, correlating the vocalization-preparatory phase with external social events could reveal stimuli that fail to reach a threshold for an audible response, but might still have a stimulatory effect on the receivers’ motivation to reply. On the flip side, partial vocalization events, in which an individual was interrupted before the active vocalization phase, could be detected. Both of those conditions have the potential to be reflected in respiratory changes typical to vocal preparation, but not resulting in call emission.

To identify VCRMs, we observed meerkats [*Suricata suricatta* (Schreber 1776)], a mammalian species with very well understood social and communication systems (Clutton-Brock and Manser, 2016; Manser et al., 2014). Meerkats are social mongooses, living in the Kalahari Desert region of Southern Africa. They have an extensive vocal repertoire and most of their behaviours are accompanied by vocalizations (Gall and Manser, 2017; Manser, 1999; Townsend et al., 2010). This species has been intensively studied for more than 20 years and the wild meerkat population, monitored by researchers at the Kalahari Research Centre (KRC), South Africa, is well habituated to human presence (Clutton-Brock and Manser, 2016). Close access to the animals allows individual audio recording and a clear distinction between calls produced by the recorded focal individual and the calls produced by neighbouring conspecifics (Demartsev et al., 2018).

While individual audio recording is extensively applied in field studies, respiration monitoring is less trivial. Solutions for externally tracing respiration, outside of laboratory settings, mostly rely on wearable sensors, detecting rib cage expansion and contraction movements (Chu et al., 2019). A non-invasive alternative for recording respiration is based on thermographic imaging of external airway openings (Pereira et al., 2016). The changes in surface temperature of the airways are due to the temperature differences between the inspired and expired air; under most circumstances, cooler, drier air from the environment is inhaled and warmer, moist air from the lungs is exhaled (Pereira et al., 2019). These temperature modulations are shown to reliably represent respiration and correlate not only with the respiration waveform, but also with tidal volume (Vainer, 2018).

In this work, we present the methodological procedures for recording respiration using thermographic imaging of non-restricted subjects in natural settings. We demonstrate the method's sensitivity for detecting VCRMs and discuss the potential for identifying call intention by breathing patterns as well as the effect of conspecific calls on the respiration rate of focal individuals.

## MATERIALS AND METHODS

### Field procedures

Data were collected in June 2019 at the Kalahari Research Centre, in the Kuruman River Reserve, Northern Cape, South Africa. Daily, at dawn, one meerkat group was observed during its morning emergence from the sleeping burrow. Following emergence, meerkats often spend time in a bipedal posture, warming up in the sun before beginning to forage. This behaviour is accompanied by the emission of ‘sunning calls’, a short and soft call type that potentially functions as a social bonding signal (Demartsev et al., 2018). During this time window, a focal animal, located at >0.5 m to the nearest neighbour, was simultaneously audio recorded and filmed. The focal subjects were not restricted in their movement and were able to freely move, change location and posture. Each continuous individual recording lasted up to ∼5 min. The daily recording session was terminated after the majority of the group moved away from the sleeping burrow and started foraging.

The focal animal's vocalizations were recorded using a Marantz PMD-661 solid state digital recorder (Marantz, Japan) and a directional Sennheiser ME66 microphone with K6 power module (Sennheiser electronic, Germany), sampling rate 44.1 kHz, 16-bit. A FLIR T1030 (FLIR Systems, USA) thermal camera with 12 deg lens was fixed on a tripod, in front of the focal animal at 1–1.5 m distance. The camera was angled in a way to not obstruct the animal's field of vision while having a horizontal view of its nasal region. The audio recording and thermal video data were collected simultaneously for each individual. To achieve synchronization between the audio and video streams a piezo-electric lighter was activated 3 times in front of the camera and in close proximity to the microphone. The distinctive sound coupled with the appearance of heat signature provided the markers required for accurately aligning the audio and video tracks.

All procedures were approved by the ethical committees of the University of Pretoria, South Africa (permit: EC011-10) and the Northern Cape Department of Environment and Nature Conservation (permit: FAUNA 1020/2016).

### Audio and video processing and synchronization

Collected audio files were analysed in Avisoft-SASLab Pro v. 5.2.13 (Avisoft Bioacoustics, Germany) and all recorded calls were manually marked. Focal (recorded individual) and non-focal (NF; conspecific neighbours) calls were identified by their relative amplitude levels and labelled accordingly (Fig. S1). Recordings with no clearly distinguishable focal versus non focal differences in amplitude were omitted from further analysis.

FLIR video (CSQ) file formats were converted to AVI (FFMPEG) format while retaining the original frame rate, pixel dimensions and colour palette. The conversion was performed using ThermImageJ plugins for the Fiji (ImageJ) environment (https://github.com/gtatters/ThermImageJ) and carried out as outlined in Tattersall et al. (2020). The conversion process first split the video file into its subsequent FLIR file format (FFF) image frames using a custom perl script (available in ThermImageJ; https://github.com/gtatters/ThermImageJ). The underlying raw thermal data from each FFF file was extracted using Exiftool's (https://exiftool.org/) raw thermal image extraction option, which created a series of lossless greyscale JPEG-LS files, where pixel intensity corresponded to the raw sensor data from the thermal imaging camera. These files were subsequently converted into a portable video file (AVI) using FFMPEG software and used for synchronization with the audio recordings. Matching audio (WAV) and video (AVI) files were imported into SHOTCUT (Meltytech, LLC, USA) video editing software. The synchronization markers in both audio and video tracks were manually aligned and a conversion table between video and audio time lines was generated. No time drift between the AV tracks in different parts of the recording was detected and single triplet of synchronization markers was sufficient for the duration (≤5 min) of the recorded files Movie 1).

### Nasal area detection and tracking

For locating the animal's nasal area in the videos, the relevant ROI (region of interest) on each video frame were defined and tracked. To automate ROI tracking, Loopy (loopbio, Austria) pose estimation tool was used. Briefly, a key point detector was trained by manually labelling 1400 exemplars of meerkat nostril centre points, randomly pulled from our video data set. This was used as the training set for a deep-learning-based detector, which was used to process all video files and list the pixel coordinates for each detection above 0.95 certainty score. Five randomly selected video files were visually inspected for correct ROI detection (Movie 1).

### Nasal temperature estimation

Raw nostril detection output consisted of frame number, *x–y* pixel coordinates and indication of laterality (left versus right nostril). To extract intensity values corresponding to the raw sensor radiance value, a custom-written macro batch function was used for exporting pixel coordinates into Fiji, defining a 10×5 pixel ROI (Movie 1) and measuring its median intensity. The ROI size for intensity extraction was chosen to include the size of meerkat nostril pixel dimensions across filming distances (16×9–11×6 px). The intensity values were converted to estimated temperature by using standard thermal image conversion algorithms outlined in ThermImageJ (https://github.com/gtatters/ThermImageJ) and described in Minkina and Dudzik (2009). The *raw2temp* function in ThermImageJ follows FLIR's algorithms, requiring information on emissivity, relative humidity, atmospheric temperature, reflected temperature, object distance and the camera's unique calibration constants. An emissivity of 0.95 was assumed based on other biological studies (Tattersall, 2016), while the other parameters were recorded at the beginning and, as permitted, throughout the daily data collecting session (1–2 h). As our data analysis was focused on changes in temperature, the average atmospheric values recorded in a given day were used for temperature conversion of all videos captured on that day. This approach helped us to streamline workflow and was made possible by the relatively stable atmospheric conditions, throughout which the possible error in absolute temperature measurements would be minimal (Tattersall, 2016).

The calibration constants were extracted from the original thermal image file using Exiftool. Sample images were tested using FLIR's ResearchIR (FLIR Systems) software and compared with the functions above to ensure the extracted temperature calculations were according to manufacturer recommendations.

### Data quality control and filtering

The video data were subjected to several quality control and filtering steps. Frame sequences shorter than 150 frames (5 s) and with ROI detection gaps of more than 5 consecutive frames were omitted. This ensured that analysed segments span at least three full respiration cycles allowing for reliable identification of periodicity associated with breathing. The frame cut-off was decided based on the mean±s.e.m. meerkat respiration rate of 0.603±0.112 Hz (Worthington et al., 1991) and the 30 frames s^−1^ rate of the recorded videos (3 respiration cycles×1.6 s cycle^−1^×30 frames s^−1^=144 frames). Only frame sequences with simultaneous detections of both left and right nostrils were used to control for focal subject head orientation as front-view, minimizing potential measurements errors due to varying angles of ROI exposure. Only sequences with clearly identifiable cyclic patterns were selected for further analysis (Fig. S2A,B) by visual inspection of time–temperature line plots.

### Data smoothing and respiration phase detection

A low-pass Butterworth filter (second order, critical frequency=1/5) was applied for smoothing the high frequency noise in temperature values, possibly originating from muscle contractions affecting ROI shape and tracking errors (Fig. S2B). A custom script was used to locate local minimum points, indicating maximum cooling of the nostril, equivalent to the end of inspiration phase. The local minimum points were used as a starting point for locating the beginning of the inspiration and the end of expiration phases. This was achieved by calculating the local slope of the curve to the point of it becoming infinitesimal, indicating the plateauing of the curve and the boundaries of expiration pause (Fig. S1A, Fig. S2C) – a rest phase in which the nostril temperature is expected to remain stable. Identification of the three transition points (end of expiration pause, maximum inspiration, maximum expiration) defined a single respiration cycle and allowed us to quantify each respiration phase (inspiration, expiration, expiration pause) in terms of duration (s), amplitude (Δ°C) and slope (amplitude/duration).

### Marking vocalization times and defining breath types

**Fig. 1.**
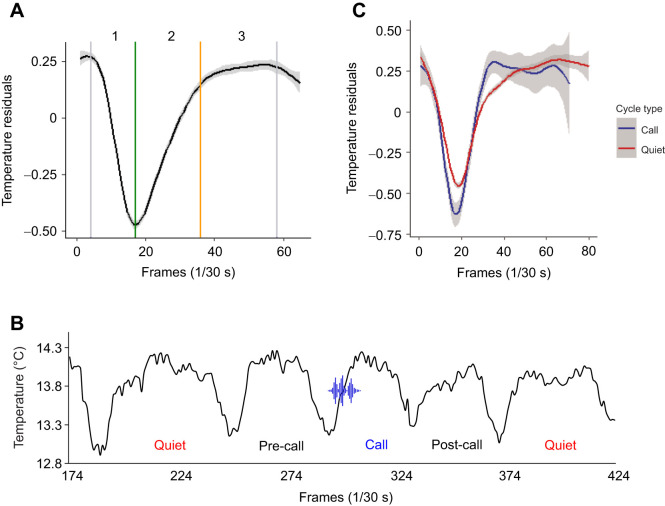
**Respiration phases and types of respiration cycles.** (A) Mean meerkat respiration cycle (*n*=283), as estimated from nostril temperature traces. Phase 1: inspiration; phase 2: expiration; phase 3: expiratory pause. Coloured vertical lines mark phase transition points: dark green, maximum inspiration point; orange, beginning of the expiration pause; grey, end of expiration pauses and the starting point of active inspiration. *x*-axis is video frame rate set at 1/30 frames s^−1^. *y*-axis is ‘residual temperature’ calculated by subtracting per frame measured ROI temperature from mean ROI temperature of the whole analysed segment. (B) Schematic visualization of respiration cycle types. Call, a cycle in which focal call was recorded; Post-call, cycle following call cycle; Pre-call, cycle preceding call cycle; Quiet, respiration cycle not associated with vocalization. The sound wave icon (designed by macrovector/Freepik) indicates the phase in which audible vocal production is taking place. *x*-axis is video frame rate set at 1/30 frames s^−1^. *y*-axis is estimated temperature of ROI. (C) Comparison of mean Call (*n*=32) and Quiet (*n*=192) respiration cycles. The curves are mean residual temperature values. Shaded areas are 95% CI. The curve shapes show a higher amplitude during the active respiration phase and shorter expiration pause of Call cycles.

**Fig. 2.**
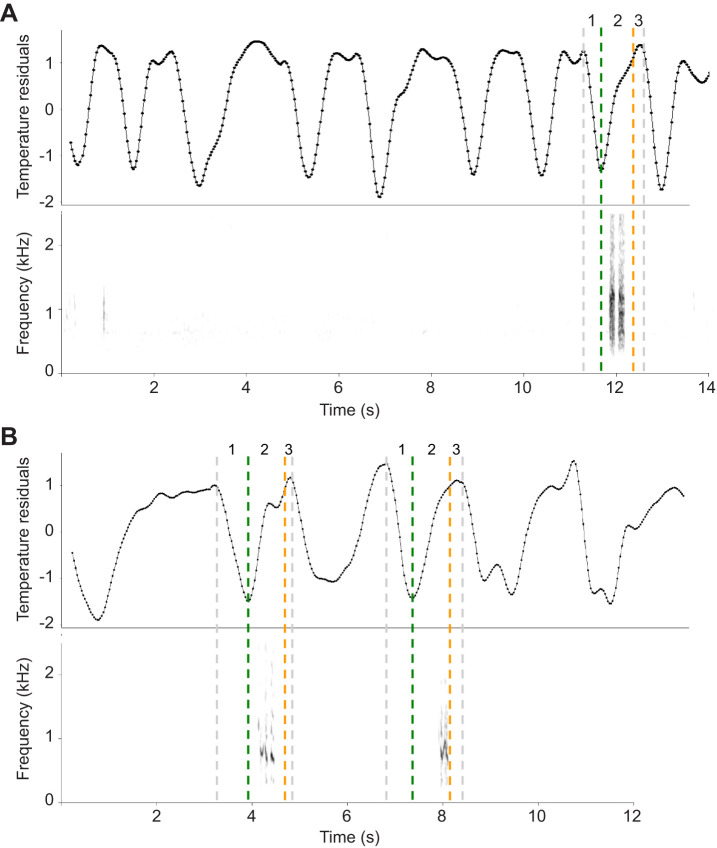
**Examples of continuous respiration traces plotted above the spectrogram of the corresponding audio recording.** (A,B) Two different respiration sequences during which calls were produced. Breathing phases and transitions are marked on the respiration cycle corresponding with call emission. Breathing phases: 1, inspiration; 2, expiration; 3, expiratory pause. Phase transition points: dark green, maximum inspiration point; orange, beginning of the expiration pause; grey, end of expiration pause and the starting point of active inspiration. The call timing overlaps with the exhalation breathing phase as it is typical for most mammalian vocalization. Breathing cycles associated with vocal emission are demonstrating irregularities in comparison to Quiet respiration.

Video frames corresponding to focal and non-focal (NF) call times were marked according to the audio-video synchronization table and breathing cycles were defined as: (1) Call: cycles during which focal calls were produced; (2) Pre-call: cycles immediately preceding a call cycle; (3) Post-call: cycles immediately following a call cycle; (4) Quiet: cycles which were not associated with vocal production (Fig. 1B, Fig. 2). In order to ensure correct identification of respiration cycles located on the edges of detected breathing sequences, audio recordings were examined for focal calls occurring 2 s before and after usable video sequences. If focal calls were recorded within those time windows, the designation of the following or preceding cycle was set as Post-call or Pre-call, respectively.

Additionally, video frames corresponding to breath phases (inspiration, expiration, etc.) were defined according to the timing of NF calls; NF heard: phases during which or immediately preceding when a NF call was produced; No calls: breathing phases not associated with NF calls.

### Data structure summary and statistical analysis

The collected raw data set included ∼3 h of AV material of 33 adult individuals from 3 different social groups. Approximately 25% of the raw material was omitted from further analysis at the quality control phase due to acoustic interferences and inability to reliably identify focal calls. Individuals that did not produce any calls were discarded from the dataset to allow within-individual data permutation procedure. Each traced respiration cycle was divided into three breathing phases (Inspiration, Expiration, Expiratory pause) which were tested separately. Following the processing and filtering stages, a final dataset consisted of 800 respiration phases (inspiration=256, expiration=283, expiratory pause=261) defined by the detection of vocalization: (Pre-call=78, Call=86, Post-call=88, Quiet=548). The per-individual call count mean±s.d. in the analysed data set was 2.9±2.4 calls from 10 individuals (4 males and 6 females). The test statistic used was the change in the mean (Δmean) of the measured parameters (e.g. mean duration of inspiration phases in Call cycles–mean duration of inspiration phases in Quiet cycles).

A permutation procedure was designed in which respiration cycle type identifiers (e.g. Call and Quiet) were randomly reshuffled against the measured values creating a new null data set. To control for potential biases related to variation in respiration and call rates between individual animals we restricted the permutations to within individuals only. Permutations were repeated 10,000 times, calculating the Δperm_mean for each iteration. Two-tailed pseudo *P*-values were estimated by calculating the percentage of absolute Δperm_means, which were lower than absolute Δmean (calculated from the data).

For estimating the effect of neighbour calls on individual respiration, only ‘Quiet’ respiration cycles were subsetted from the data, as an individual’s own call emission could potentially mask the likely subtle effects of auditory perception. Based on the timing of NF calls, breathing phases during which or immediately preceding which NF calls were detected were designated as ‘NF heard’. The analysed dataset consisted of 714 respiration phases (NF heard=142; No calls=572). The dataset was analysed using a similar permutation procedure, reshuffling ‘NF heard’ and ‘No calls’ respiration phase designations.

**Fig. 3.**
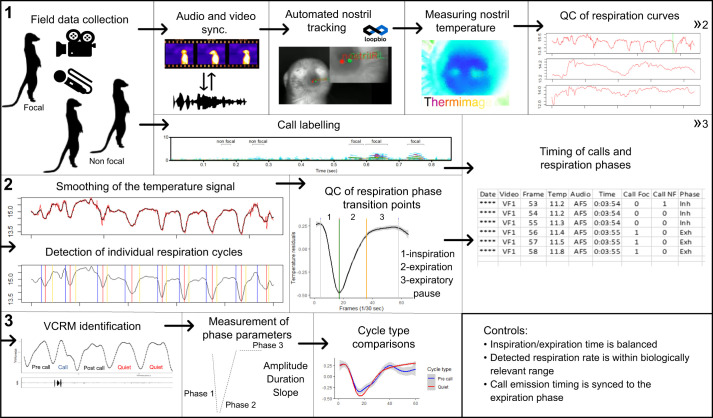
**A schematic of the data collection, processing and analysis process.** (1) Meerkats are recorded and filmed with thermal video camera. Audio and video tracks are synced by aligning audio and heat signature cues. Calls are labelled with distinction between calls of a focal individual and calls of neighbouring conspecifics. Meerkat nostrils are tracked using Loopbio posture detection tools. Using the tracked nostril ROI coordinates, nostril median temperature is calculated from the image intensity, using ThermImageJ plugins. The temperature traces are plotted on a time scale and visually inspected for clear continuous cyclic patterns. (2) The selected respiration curves are smoothed and go through a local peak detection process for identifying respiration phase transition points. The detection of respiration phases (inspiration, expiration, expiratory pause) is visually verified. A summary data set is constructed including ROI median temperature values, respirational phase, video frame numbers, corresponding audio time, timing of focal calls, timing of non-focal calls. (3) Respiration cycles are labelled according to association with produced calls: Pre-call, preceding call production; Call, call produced within the cycle; Post call, following call production; Quiet, no association with vocal production. Three measurements are calculated for each respirational phase: amplitude, temperature change; duration, length of the phase; slope, change of temperature for a unit of time. A pairwise comparison is performed with vocalization-associated respiration cycles compared with Quiet cycles. QC, quality control; VCRMs, vocalization-correlated respiratory movements.

All quality control, filtering procedures, permutations and statistical testing were done in R 3.6.3 (https://www.r-project.org/). A graphical representation of the data collection, processing and analysis process is detailed in Fig. 3.

## RESULTS

**Fig. 4.**
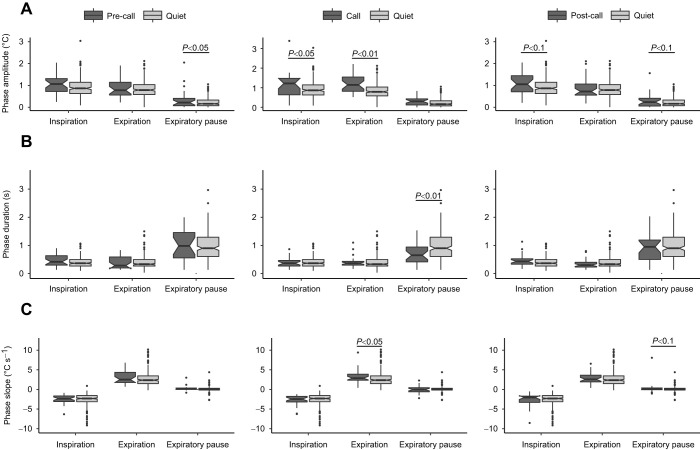
**Parameters measured and compared for the different respiration phases per cycle type.** Horizontal brackets denote pseudo *P*-values calculated in pairwise permutation procedure detailed in the Materials and Methods. Pairs without brackets do not show significant differences. Sub-panels show pairwise comparison of: Pre-call/Quiet (left), Call/Quiet (middle) and Post-call/Quiet (right) cycles. Respiration phases (inspiration, expiration, expiratory pause) are plotted on the *x*-axis. Boxplots show median, IQR (box) and 95% CIs of the median (notches). (A) Temperature (°C) change during respiration phase. Expiratory pause phases (*n*=26) of Pre-call cycles show a tendency towards higher change in temperature in comparison to Quiet respiration cycles (*n*=177). Call cycles show significantly higher temperature change during both inspiration (*n*=25) and expiration phases (*n*=32) in comparison to respective phases in Quiet cycles (*n*=179 and 192). (B) Duration (s) of the respiration phase. Duration of expiratory pause phase (*n*=29) of Call cycles are significantly shorter in comparison to Quiet respiration cycles (*n*=177). (C) Slope of the respiration curve per relevant phase. Expirations (*n*=27) during Pre-Call cycles show a tendency towards higher slope values in comparison to Quiet respiration cycles (*n*=192). Call cycles show significantly higher slope values during expiration phases (*n*=32) in comparison to Quiet cycles (*n*=192).

The nasal region of recorded subjects, tracked in the collected thermal video material, showed an obvious variation in median temperature corresponding to respiration trace (Movie 1). Examination of the respiration cycle ratio (inspiration time/expiration time) showed a nearly symmetrical value of 0.987, indicating a roughly balanced airflow during active respiration (Fahlman and Madigan, 2016) and confirming consistency of our respiration tracing. As expected, vocalization demonstrated detectable respiratory exertion (Fig. 1C, Fig. 2). The amplitude (Δtemperature) of the expiration phase during Call cycles (median=1.14, IQR=0.73) was significantly higher than in Quiet cycles (*P*<0.01, median=0.78, IQR=0.40, Fig. 4A, middle panel, Fig. S3A). The slope (temperature–time) of expiration was also significantly steeper (median=2.87, IQR=1.52) in comparison to the control condition of Quiet cycles (*P*=0.02, median=2.33, IQR=1.56, Fig. 4C, middle panel, Fig. S3C). These results correspond to higher aerobic expenditure during call emission, which confirms the accuracy and sensitivity of the proposed methodology. This justified testing the ability for detecting respiration patterns associated with the preparation to call and the effects of conspecific vocal signals on focal respiration.

Median inspiration amplitude in Call cycles (median=1.21, IQR=0.83) was larger than in Quiet cycles (*P*=0.02, median=0.87, IQR=0.48), indicating that calling requires deeper inspiration (Fig. 4A, middle panel, Fig. S3A). Comparison between Pre-call and Quiet respiration cycles (Fig. 1B) showed a difference between expiratory pause amplitudes of Pre-call (median=0.22, IQR=0.33) and Quiet cycles (*P*=0.04, median=0.16, IQR=0.26, Fig. 4A, left panel, Fig. S4A). Taken together, this supports the prediction that there are detectable alterations of respiration patterns before call emission, with the most noticeable changes occurring during the inspiration phase of the Call respiration cycle; however, Pre-call cycles are also potentially affected. The recovery from vocalization is evident from the duration of the expiratory pause of Call cycles (median=0.65, IQR=0.53), which demonstrates a shorter rest period following call emission in comparison to Quiet respiration cycles (*P*<0.01, median=0.9, IQR=0.65, Fig. 4B, middle panel, Fig. S2B). Examination of Post-call cycles shows traces of recovery with trends for higher amplitude during inspiration and expiratory pause in comparison with Quiet respiration cycles (Fig. 4, Fig. S5, Table 1).

**Table 1.**
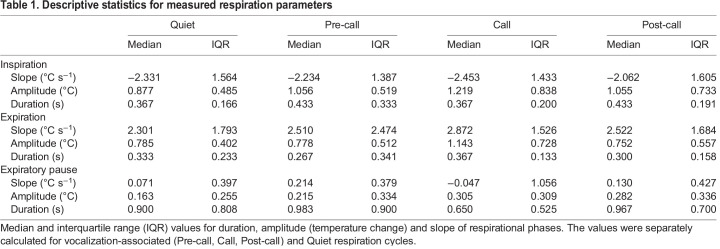
Descriptive statistics for measured respiration parameters

To test whether the magnitude of vocalization-associated respiration changes are correlated with the duration of produced call; parameters (amplitude, duration, slope) of Call cycles were subtracted from the corresponding median values of Quiet cycles. A Spearman correlation test, between call duration and the difference from median of the three respiration phases was performed. No significant correlation with call duration could be detected (Table 2).

**Table 2.**
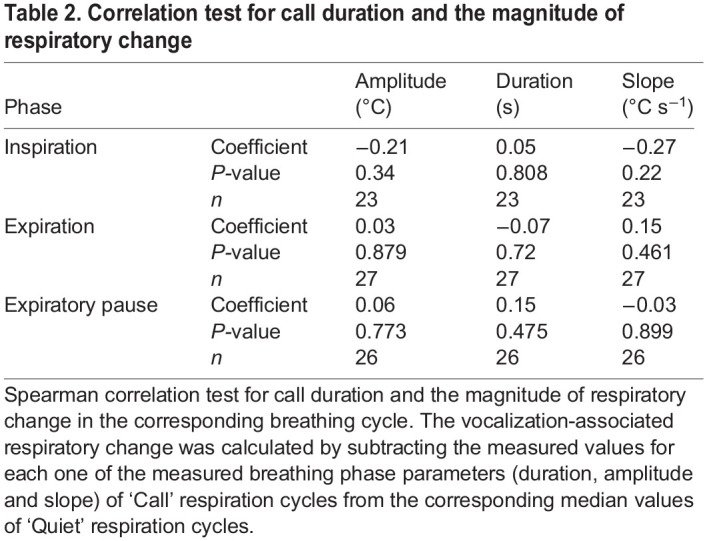
Correlation test for call duration and the magnitude of respiratory change

**Fig. 5.**
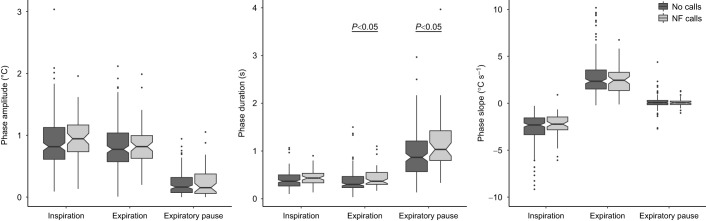
**Parameters measured and compared for the three respiration phases of Quiet respiration cycles with (non-focal calls) and without (no calls) conspecific calls.** Horizontal brackets denote pseudo *P*-values calculated in pairwise permutation procedure detailed in the Materials and Methods. Pairs without brackets show no significant differences. Boxplots show median, IQR (box) and 95% CIs of the median (notches). Measured parameters are: amplitude, temperature (°C) change during respiration phase; duration, length (s) of the respiration phase; slope, change in temperature over the duration of the respiration phase. Duration of inspiration (*n*=75) and expiration (*n*=54) phases of the focal are longer when non-focal (NF) calls are heard in comparison to respective phases of ‘No-call’ cycles (*n*=238 and 291).

It has been previously hypothesized that conspecific calls have the potential to stimulate, but also suppress focal vocalizations (Demartsev et al., 2018). Thus, we tested whether focal respiration patterns were affected by incoming non focal calls as a potential indication of changes in motivation towards vocal production. In a comparison between ‘NF heard’ respiration phases (during or immediately before which NF calls were recorded) and ‘No calls’ phases (no NF calls were recorded), the durations of expiration and expiratory pause, in the former, were significantly longer (*P*≈0.01) and inspiration phase demonstrated a similar trend (*P*=0.08, Fig. 5B, Fig. S6B). This indicates a transient decrease in breathing rate when conspecific calls are heard by the focal. Additionally, the inspiration and expiration slopes of the NF heard respiration phases appeared steeper; however, they did not reach statistical significance (*P*≈0.06, Fig. 5C, Fig. S6C).

## DISCUSSION

This work describes a methodological framework for thermography-based remote monitoring of breathing in a free-ranging mammal and demonstrates the sensitivity of this method for detecting vocalization-correlated respiratory movements (VCRMs). Additionally, it demonstrates the capability to measure the effects of conspecific calls on the respiration of a focal subject as a potential indication of attention and signal perception.

As expected, calling had an effect on respiration (Fig. 1C), and despite the relatively low amplitude and short duration of the calls in focus (50 ms, Fig. S1), they could not be accommodated by regular expiration. Both the amplitude (Fig. 4A) and the slope (Fig. 4C) measurements during calling exceeded expirations while quiet, probably because of the increase in subglottal pressure needed for call production. The duration of an expiratory pause (in which the volume of lungs remains unchanged, Fig. 1A) is dependent on accumulation of the respiratory chemical drive (low oxygen and high carbon dioxide levels) leading to the activation of respiratory muscles and active inspiration (Rafferty et al., 1995). Additionally, mechano-receptors located in the lungs and the airways, react to lung inflation by adjusting the rate and volume of respiration. Vocalizing increases the gas flow during the expiration phase, resulting in a decreased lung volume in comparison to quiet respiration. This is expected to increase the respiratory drive, and to shortening the expiratory pause. Our results fit this assumption, as the duration of call cycle expiratory pause is significantly shorter than during quiet respiration cycles (Fig. 4B). Taken together, the increased flow during vocalizing and the shortening of subsequent expiratory pause support the notion that the temperature measurements, extracted from the subject's nasal area are representative of its respiration curve. The resolution and consistency of this representation were adequate for detecting the magnitude of changes related to vocal emission. The proposed methodology may be limited in its ability to detect mini-breaths, associated with separate notes in trill vocalizations, for example. However, the effect of the whole calling bout on the full tidal volume trace is likely to be detectable. This procedure can be extended to other mammalian species given the possibility of individual audio recording and steady horizontal view of the nasal region for the duration of at least three full respiration cycles. Special attention should be given to environmental conditions during thermal data collection, as with ambient temperature being nearly equal or above the subjects’ body temperature, the spectral resolution between inspired and expired air will be limited.

The changes in respiration preceding vocal production are of interest as they indicate preparation for vocalizing, either as part of an automated process (Riede et al., 2020) or perhaps having an intentional component. Mammalian vocal production is usually considered to be a flexible behaviour both in terms of call production and usage (Maciej et al., 2013; Sugiura and Masataka, 1995). Recent findings suggest that there is no point of no-return for calling and demonstrate that some species can suppress calls shortly before and even during call emission (Demartsev et al., 2018; Pomberger et al., 2018). In social settings, there could be instances in which an individual, stimulated to vocalize, was interrupted immediately before sound emission. The respiration patterns preceding calling in the inspiration phase of call cycles (Fig. 4A) and trends visible in expiration and expiratory pause of the pre-call cycles (Fig. 2A,C), suggest that the window of preparation to call is detectable. The physiological duration of this window is likely to be equal to the duration of inspiration, corresponding to the interval between the neural activation of vocalization and accumulating the pressure capacity for vocal emission. This theoretical minimum, however, is likely to be affected by the length of the call and by the intensity of the vocal exchange. Emission of long call or call sequences, performed on a single respiration cycle requires deeper inspiration (Riede et al., 2020). The current data could not show correlation between the duration of the produced call and the magnitude of the detected respiration changes. The described methodology is, however, fully capable of detecting such effects, perhaps if comparing different call types of variable duration or looking at continuous vocal bouts. Similarly, louder vocalizations requiring higher subglottal pressure could also result in stronger VCRM deviation from quiet respiration. To what extent respiratory preparations for longer or louder calls are distinguishable and whether call types or calling properties can be independently predicted from preceding respiration patterns is yet to be determined.

Precise call timing, needed to achieve caller coordination for synchrony or anti-synchrony, might result in multiple calling initiation attempts and suppression events, which could be reflected in irregularities of respiration patterns. By tracing focal respiration during exposure to conspecific calls we could detect social effects on quiet breathing cycles, specifically with longer expirations and expiratory pauses (Fig. 5). Although, experimental work is needed to test the causal link between conspecific calls and focal respiration, we can think of three potential explanations for the elongation of respiration phases. First, ‘sunning’ call exchanges in meerkats demonstrate strong turn-taking patterns (Demartsev et al., 2018) and individuals time their calls between the calls of multiple conspecifics, perhaps to avoid overlap. This could result in failed initiations and transient irregularities in respiration, as each suppressed calling initiation event could be represented by a partial respiratory preparation for calling. Second, the elongation of breathing phase duration could be a general indication of attention. It was suggested that attention and respiration are coupled (Maric et al., 2020; Melnychuk et al., 2018) and that respiratory inhibition is related to increased auditory sensitivity (Stekelenburg and Van Boxtel, 2001). So, tracing respiration during social vocal interactions could aid in identifying momentary attention changes that do not manifest into behavioural responses, perhaps in combination with heart rate logging and muscular micro movement detection. Third, slowed down breathing could be related to the proposed function of meerkat sunning call exchanges as an acoustic grooming behaviour. Similarly to direct physical grooming, acoustic grooming is expected to have an appeasing effect on the participants (Kulahci et al., 2015), which can result in physiological responses of decreased heart rate and slower breathing. Further work is required in order to distinguish between ‘vocalization attempts’, ‘auditory perception’ and ‘general appeasing’ explanations for the demonstrated slowing down of breathing.

Examining an animal's VCRMs in producing long, loud, high pulse rate or highly modulated calls offers interesting perspectives for understanding the production similarities with the sequential assembly of vocal utterances in human speech (Hernandez et al., 2017). The ability for complex respiration control has been suggested to be a uniquely human trait, likely important for syntax and combinatorial structure of human speech (MacLarnon and Hewitt, 1999). These claims, however, were based only on mostly ancillary data, as direct respiration monitoring during vocal production of animal subjects is scarce. Existing evidence indicate that a relationship between breathing movements and vocal production is often more complex than the call-per-breath paradigm (Hage et al., 2013; Häusler, 2000) and there is little support for the claims that animals have limited ability to control pitch, modulate amplitude and produce rapidly changing sound sequences on a ‘single breath’ (Fitch, 2018; Maclarnon and Hewitt, 2004). There are numerous mammalian species capable of long, variable and high-rate vocal performance (Haimoff, 1984; Passilongo et al., 2010), which could benefit from efficient respiration control beyond the call-per-breath paradigm. Additionally, there is a growing body of evidence demonstrating precise motor control of animals over both initiation (Hage and Nieder, 2013) and termination (Pomberger et al., 2018) of their vocal signals. Further focused studies on animal respiration during vocal production would be able to refute or confirm the claims about complex respiration control being an adaptation to the complexity of human vocal production.

This work provides the methodological framework for remote respiration monitoring in free-ranging animals and demonstrates its sensitivity for detecting respiration cues indicating vocal preparation. Further exploration of the association between respiration patterns and vocalization will provide a perspective on intentionality and planning of animal vocal signalling. Additionally, respiration cues could function as an interaction regulation mechanism, with individuals identifying their neighbours’ preparations to vocalize and adjusting their own behaviour accordingly. Addressing this aspect could provide insights on the levels of social awareness during communicative interactions and the ability to perceive conspecific intentions.

## Supplementary Material

Supplementary information
