# Supplementary material for: Vocalization-associated respiration patterns: thermography-based monitoring and detection of preparation for calling
Source: J Exp Biol. 2022 Mar 10;225(5):jeb243474. doi: 10.1242/jeb.243474 (PMC8976942; doi:10.1242/jeb.243474)
Supplement: Supplementary information [file jexbio-225-243474-s1.pdf]

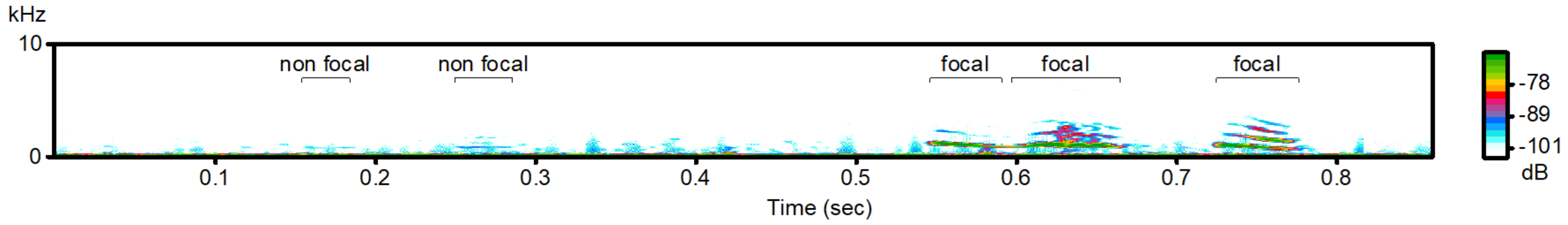

Fig. S1. Sample spectrogram with marked focal and non focal short note meerkat calls. The amplitude differences were used to distinguish focal calls from calls of distant conspecific.

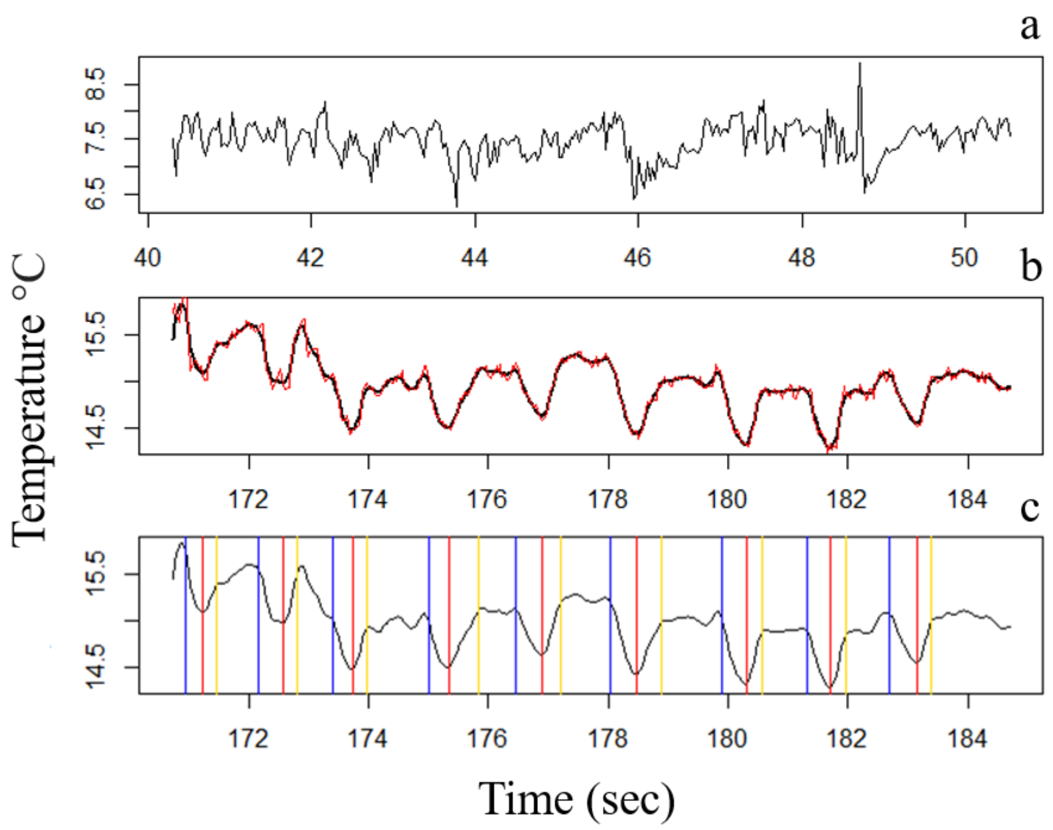

Fig. S2. Mean nostril temperature over time. a - Sample temperature curve in which clear cyclic patterns could not be identified and were omitted from further analysis. b - Sample temperature curve with clear cyclic pattern typical to respiration. Red curve is the raw temperature data. Black curve is the temperature data after applying Butterworth filter for smoothing high frequency noise. c - Filtered temperature curve with respiration phase transition points (as detected by the peak detection script) marked. Red lines are marking maximum inspiration points. Yellow lines mark the end of the active expiration phase and beginning of the expiration pause. Blue lines are marking the end of expiration pause and the starting point of active inspiration.

Journal of Experimental Biology • Supplementary information

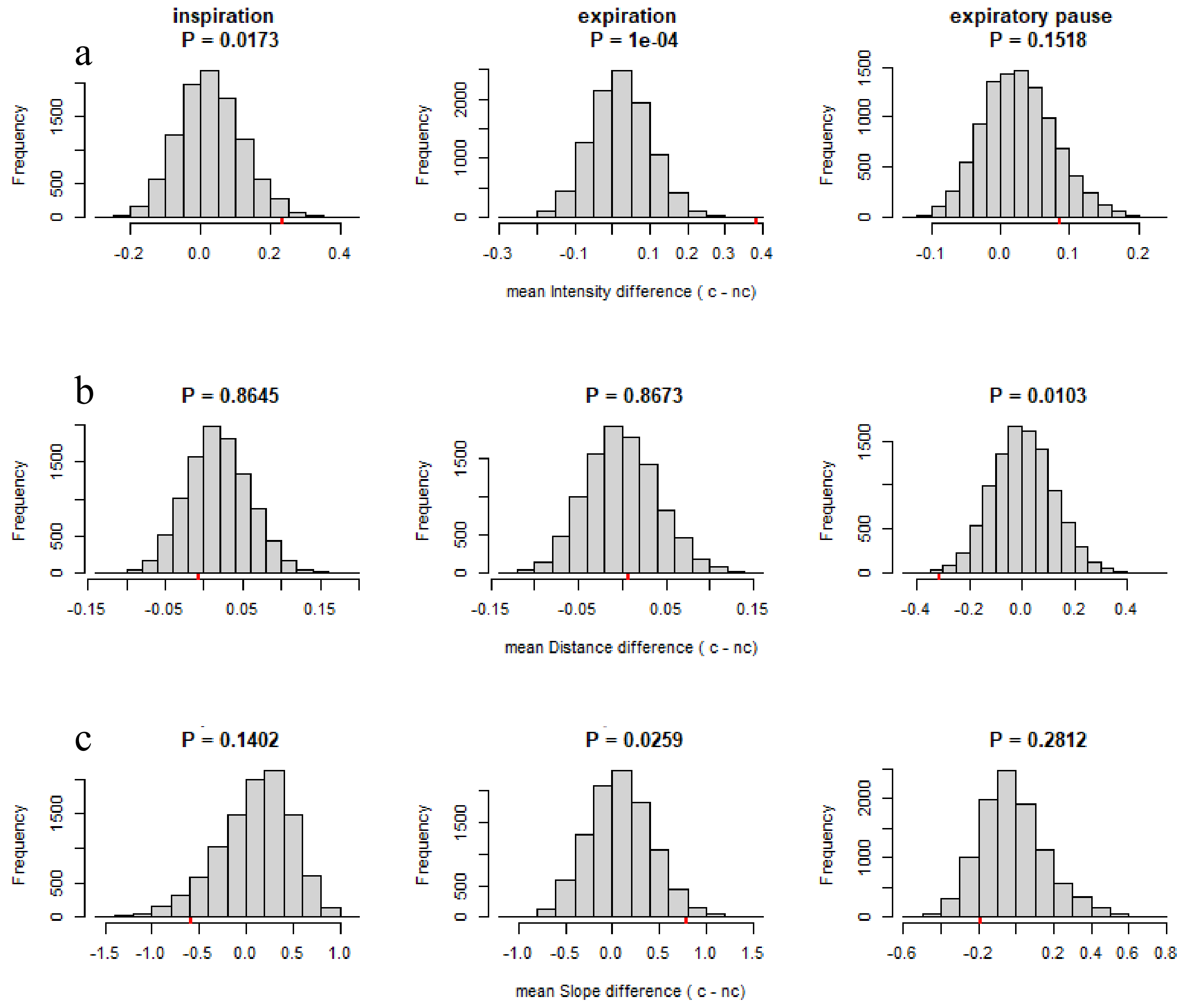

Fig. S3. Distribution of 10000 mean difference values from permutations of Quiet respiration and Call cycles. Red notch indicating the value calculated by subtracting mean measurement of Call cycles from mean measurement of Quiet cycles. P-value estimation calculated from the proportion of absolute values of the permuted null data being smaller than the difference derived from the data. a - difference in respiration phase amplitude (temperature change). b – differences in respiration phase duration. c – difference between respiration curve slopes.

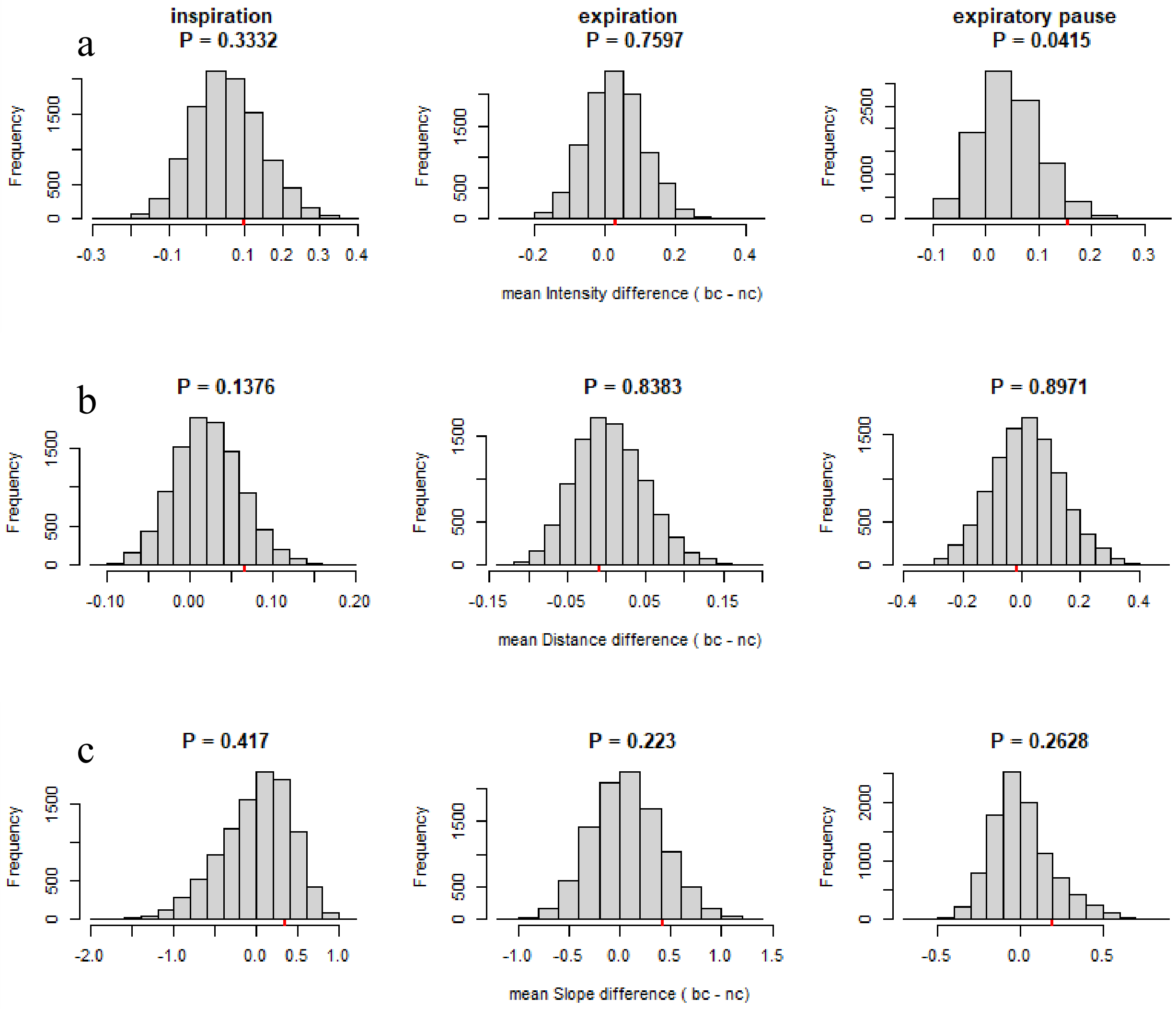

Fig. S4. Distribution of 10000 mean difference values from permutations of Quiet respiration and Pre call cycles. Red notch indicating the value calculated by subtracting mean measurement of Pre call cycles from mean measurement of Quiet cycles. P-value estimation calculated from the proportion of absolute values of the permuted null data being smaller than the difference derived from the data. a - difference in respiration phase amplitude (temperature change). b –differences in respiration phase duration. c – difference between respiration curve slopes.

Journal of Experimental Biology • Supplementary information

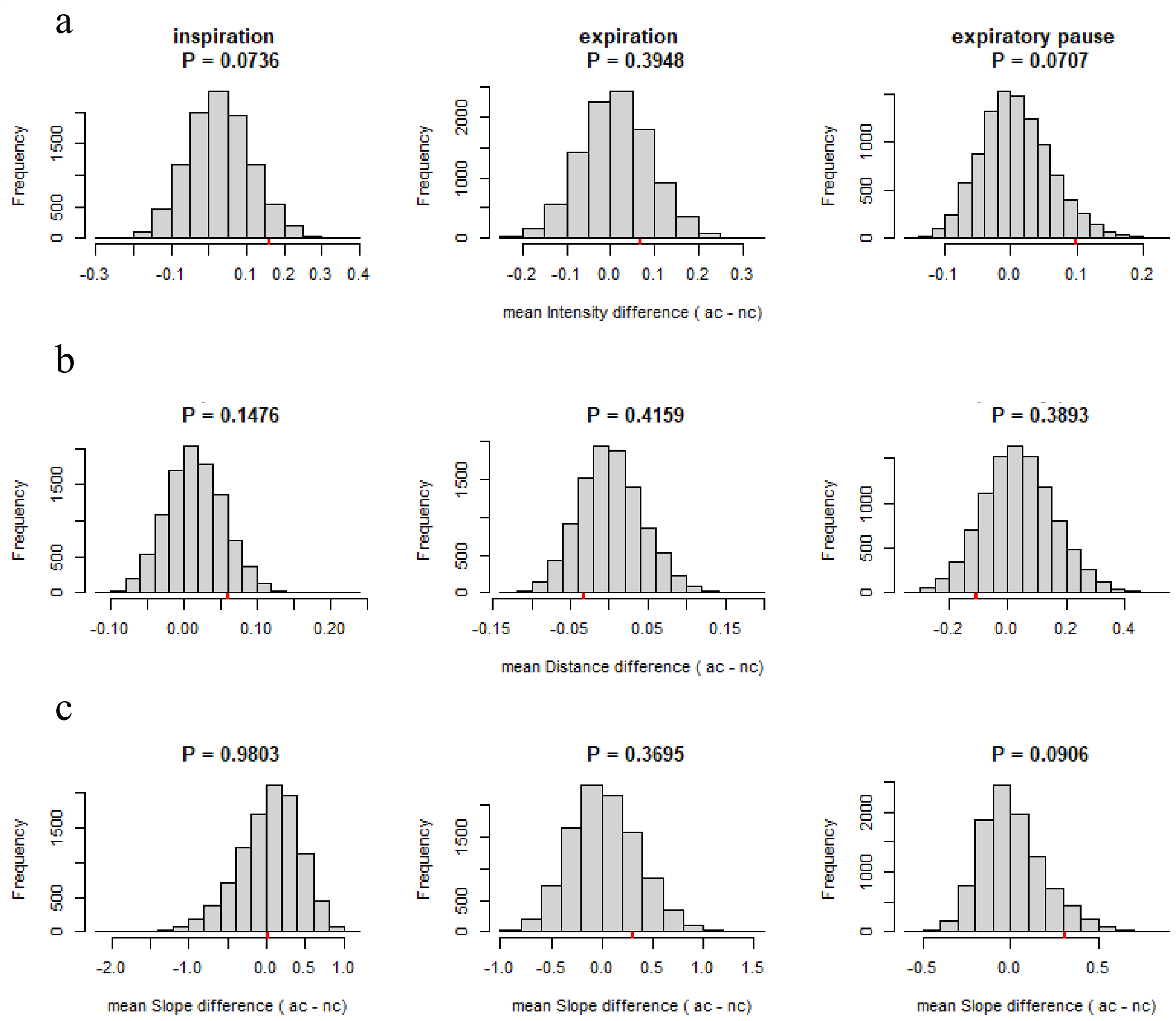

Fig. S5. Distribution of 10000 mean difference values from permutations of Quiet respiration and Post call cycles. Red notch indicating the value calculated by subtracting mean measurement of Post call cycles from mean measurement of Quiet cycles. P-value estimation calculated from the proportion of absolute values of the permuted null data being smaller than the difference derived from the data. a - difference in respiration phase amplitude (temperature change). b –differences in respiration phase duration. c – difference between respiration curve slopes.

Journal of Experimental Biology • Supplementary information

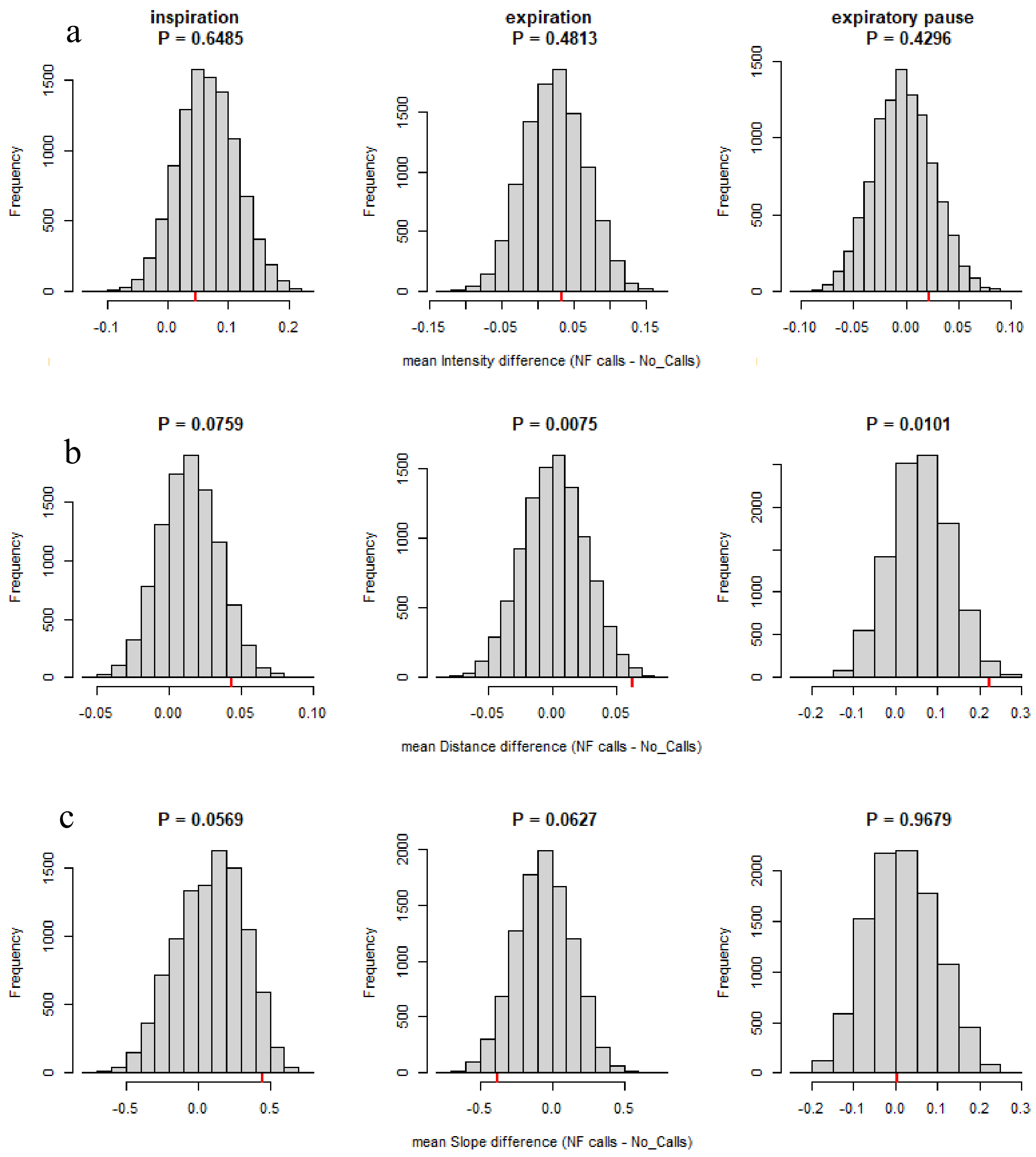

Fig. S6. Distribution of 10000 difference values from permutations of Quiet focal respirations with and without non focal calls. Red notch indicating the value calculated by subtracting mean measurement of cycles without non focal calls from mean measurement of Quiet cycles with non focal calls. P-value

estimation calculated from the proportion of absolute values of the permuted null data being smaller than the difference derived from the data. a - difference in respiration phase amplitude (temperature change). b – differences in respiration phase duration. c – difference between respiration curve slopes.

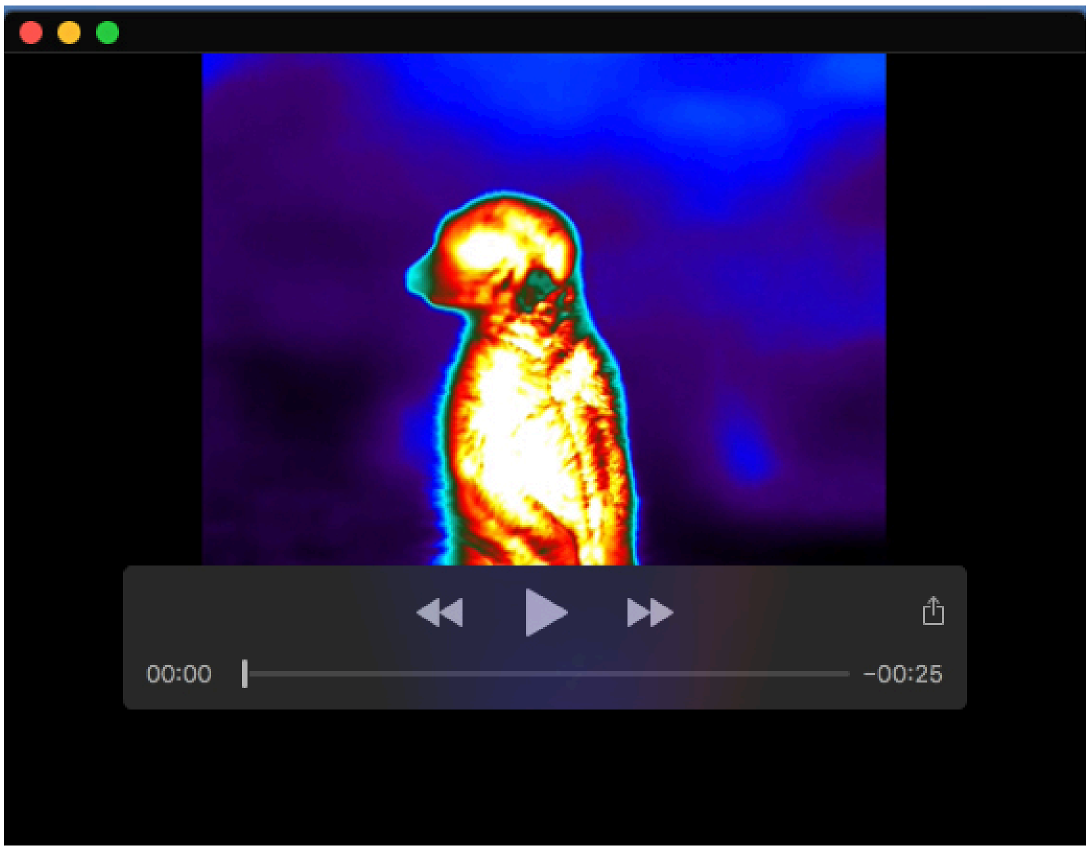

Movie 1. A vocalizing focal meerkat. Synchronized sample of thermal video and audio recording.

starting from 00:46 sec - A series of manual pings used for synchronization, slowed down to 33% of original playback speed.

starting from 01:16 - A sample of tracked nostril ROI video as outputted by Loopy pose estimation tool (loopbio gmbh, Austria). Green dot marks detections of left nostril centre. Red dot marks detections of right nostril centre.

starting from 02:00 - Sample thermal video sequence demonstrating the nasal ROI used for estimating respiration trace from changes in surface temperature. Real time temperature plot is showing the median temperature reading of the left nostril. The cyclic pattern of the temperature curve is typical to respiration. The insert shows magnified nasal area, at a slowed down playback speed, emphasizing the changes in the nostril intensity.
